# Effects of Phosphate-solubilizing Bacteria on Soil Phosphorus Fractions and Supply to Maize Seedlings Grown in Lateritic Red Earths and Cinnamon Soils

**DOI:** 10.1264/jsme2.ME22075

**Published:** 2023-05-23

**Authors:** Han Long, Jun Wasaki

**Affiliations:** 1 Graduate School of Integrated Sciences for Life, Hiroshima University, Kagamiyama 1–7–1, Higashi-Hiroshima 739–8521, Japan; 2 School of Marine Sciences and Biotechnology, Guangxi University for Nationalities, No. 188, East University Road, Nanning, Guangxi 530006, China; 3 Seto Inland Sea Carbon Neutral Research Center, Hiroshima University, Japan

**Keywords:** Cinnamon soils, Lateritic red earths, P fractions, P accumulation, Phosphate-solubilizing bacteria (PSB)

## Abstract

Phosphorus (P) is often the limiting factor for plant growth because of its low mobility and availability in soils. Phosphate-solubilizing bacteria (PSB) have been shown to increase the availability of soil P fractions, thereby promoting plant growth. We herein investigated the effects of PSB on P availability in two important Chinese soil types: Lateritic red earths (La) and Cinnamon soils (Ci). We initially isolated 5 PSB strains and assessed their effects on soil P fractions. PSB mainly increased moderately labile P in La and labile P in Ci. We then selected the most promising PSB isolate (99% similarity with *Enterobacter chuandaensis*) and examined its effects on P accumulation in maize seedlings. The results obtained showed that plant P accumulation increased in response to a PSB inoculation in both soil types and the combination of the PSB inoculation and tricalcium phosphate fertilization in La significantly enhanced P accumulation in plant shoots. The present study demonstrated that the PSB isolates tested differed in their ability to mobilize P from distinct P fertilizers and that PSB isolates have potential as a valuable means of sustainably enhancing seedling growth in Chinese agricultural soils.

Phosphorus (P), a macroelement for plants, is a limiting factor restricting their growth (Aerts and Chapin, 1999; Raghothama, 2005). Although P may be present at high concentrations in soils (Larsen, 1967), very little is directly absorbed by plants. P is mainly present in unavailable forms, namely, insoluble and organic forms. Plant and microbial functions mobilize insoluble P in soils. Since some bacteria were shown to dissolve unavailable natural raw rock phosphate in the early 20th century (Sackett *et al.*, 1908), research on phosphate-solubilizing bacteria (PSB) has been widely pursued (Khan *et al.*, 2009; Ingle and Padole, 2017). The findings obtained have demonstrated that diverse PSB isolates contribute to the mobilization of unavailable P in soils and that they support plant growth. Previous studies examined specific PSB strains and the mechanisms underlying phosphate solubilization (Ding *et al.*, 2021; Amy *et al.*, 2022). Other studies that assessed crop productivities investigated the application of PSB strains to the transformation of unavailable P in soil (Alam *et al.*, 2021; Liu *et al.*, 2021), to the supply of P to crops (Alam *et al.*, 2022; Khan *et al.*, 2022), and even to improvements in soil quality (Pathak *et al.*, 2021; Dasila *et al.*, 2022).

PSB have been shown to accelerate soil P cycling (Hafeez *et al.*, 2019; Liu *et al.*, 2022) and may counteract the antagonistic effects of soil calcification on bioavailable P (Adnan *et al.*, 2017). The PSB population may be closely related to the P fraction (He and Wan, 2022). Moreover, PSB were found to increase available P by reducing soil P retention (Halder *et al.*, 1990; Chen *et al.*, 2006; Ku *et al.*, 2018; Tian *et al.*, 2021), which has been defined as the removal of phosphate from solution by soil (Wild, 1950). The strength of P retention is mainly affected by soil mineralogy, clay content, soil pH, and climate factors, such as temperature and moisture (Batjes, 2011).

P fractions and the retention potential of soil types markedly vary worldwide. Lateritic red earths (La) and Cinnamon soils (Ci) are both important agricultural soils in China. La cover the largest surface area in China, of which approximately 350,000‍ ‍km^2^ is cultivated land, accounting for 28% of all agronomically used land in China. La has pH 5.0–5.5 and a clay content of 30–40%. It is rich in iron and aluminum oxides (He *et al.*, 2004), resulting in high P fixation to soil minerals. Moreover, La is mainly distributed in southern China in very high or high P retention potential (PRP) regions (Kochian, 2012). Cultivated lands of Ci cover a smaller area (*ca.* 20,000‍ ‍km^2^) and are mainly planted with corn and wheat. Ci is weakly alkaline, has a clay content of 20–40%, is rich in Ca (Wang, 2003), and is mainly distributed in central China and northeast China, which are moderate or low PRP regions (Kochian, 2012).

PSB exert different effects on P transformation in different soils. Hafeez *et al.* (2019) found that PSB markedly increased labile P in an alkaline (pH 8.1) sandy loam soil after fertilization with tricalcium phosphate (TCP). Delfim *et al.* (2020) reported that PSB exerted strong effects on NaOH extractable P in Andisol and Ultisol, which are both acidic soils with similar P fractions. Although these studies are informative, few comparative studies have examined the effects of the same PSB strains on P fractions in La and Ci.

In view of the above research gaps, we isolated 5 PSB strains, examined their P solubilization potential when exposed to various P sources, and assessed their effects on P fractions in unplanted La and Ci. We selected the most promising PSB isolate and investigated its effects on P accumulation in maize seedlings in both soil types.

## Materials and Methods

### Isolation and identification of PSB

Non-cultivated La and the rhizosphere soil of Chinese cabbage (*Brassica rapa* L. var. pekinensis rupr) grown in La were used to isolate PSB. One gram of each soil was suspended in 99‍ ‍mL normal saline (0.85% NaCl sterilized) solution and then gradient diluted. One hundred microliters each of the 10^–3^, 10^–4^, and 10^–5^ dilutions were spread on NBRIP agar (Nautiyal, 1999): glucose, 10‍ ‍g‍ ‍L^–1^; MgCl_2_·6H_2_O, 5‍ ‍g‍ ‍L^–1^; MgSO_4_·H_2_O, 0.25‍ ‍g‍ ‍L^–1^; KCl, 0.2‍ ‍g‍ ‍L^–1^; (NH_4_)_2_SO_4_, 0.1‍ ‍g‍ ‍L^–1^; Ca_3_(PO_4_)_2_, 5‍ ‍g‍ ‍L^–1^; and Agar, 15‍ ‍g‍ ‍L^–1^, followed by an incubation at 28°C for 5 days. Colonies with transparent circles were isolated. We enriched the isolated strains with LB at 28°C for 24‍ ‍h and then extracted DNA using the TIANamp Bacteria DNA Kit (Tiangen Biotech). Extracted DNA was used as the template for PCR amplification with the following universal bacterial primers targeting the 16S rRNA gene: 27F (Lane, 1991) and 1492R (Turner *et al.*, 1999).

PCR was performed with 1‍ ‍μL template DNA, 1‍ ‍μL (10‍ ‍μM) of each primer, 12.5‍ ‍μL 2× Taq PCR Mix (KT210; Tiangen Biotech), 9.5‍ ‍μL double distilled water, and the following steps: initial denaturation (94°C, 3‍ ‍min), denaturation (94°C, 30 s), annealing (55°C, 30 s), and extension (72°C, 1‍ ‍min). Final extension (72°C, 5‍ ‍min) was performed after 30 cycles between denaturation and extension.

PCR products were used for agarose gel electrophoresis. Gels were stained (GeneGreen Nucleic Acid Dye, RT210; Tiangen Biotech) and visualized using the Gel Imaging System (WD-9413B, Beijing Liuyi Biotechnology). PCR products were purified using a purification kit (TIANgel Midi, DP209; Tiangen Biotech). Purified PCR-amplified 16S rDNA fragments were sequenced by AuGCT. The 16S rDNA sequences obtained of the isolated strains were compared and uploaded to apply the NCBI number in the NCBI GenBank.

Identified PSB were enriched in LB at 28°C for 24 h. Bacterial cultures were centrifuged and then washed three times with normal saline to collect bacterial cells. Bacterial cells were suspended with P-free NBRIP and OD_600_ was adjusted to 0.1. PSB suspensions were used in subsequent experiments.

### Test of phosphate release ability

The phosphate release ability of PSB isolates was examined using a shake flask culture. Ca_3_(PO_4_)_2_ (tricalcium phosphate, TCP), FePO_4_, AlPO_4_, phytin (inositol hexakisphosphate and Mg and Ca salts), and lecithin (Yuanye) with the same P contents were added to P-free NBRIP. pH was adjusted to 7.0±0.2 with 0.1 M NaOH and HCl. These NBRIP derivatives were then autoclaved at 115°C for 20‍ ‍min. The PSB suspension was inoculated into sterilized NBRIP derivatives at a ratio of 1% (v/v) of culture medium. PSB was then cultured with agitation at 28°C for 5 days. A rotation speed of 150 rounds per min (rpm) was used with a rotary shaker. Each P treatment had one control without the PSB inoculation; each control and inoculation had three replicates. After the incubation, culture media were centrifuged (12,000×*g*, 10‍ ‍min) and the soluble inorganic P contents of the supernatant were measured.

### Soil viability test of PSB isolates

To examine whether the PSB isolates obtained were viable in natural soils, 2.5‍ ‍mL of the PSB suspension and 12.5‍ ‍mL of sterile distilled water were added to 50‍ ‍g of sterilized La and Ci and then incubated at 28°C in the dark for 7 days. After the incubation, 0.1‍ ‍g of soil was suspended in 99‍ ‍mL of normal saline to obtain dilutions. Fifty microliters of diluted soil suspensions was spread on NBRIP agar medium and cultured at 28°C for 5 days. Strains that formed colonies on NBRIP with transparent circles were considered to be viable in soil.

### Soil inoculation experiments

Two soils were used for inoculation experiments: La from Nanning, Guangxi, China (22°50′28.6″N 108°11′25.7″E) and Ci from Fenyang, Shanxi, China (37°17′10.0″N 111°43′11.8″E). Both soils were collected from non-cultivated lands. Soil pH (1:2.5 water), total P, and available P were 5.4, 0.6‍ ‍g P kg^–1^ soil, and 0.1‍ ‍mg P kg^–1^ soil, respectively, for La and 8.0, 0.7‍ ‍g P kg^–1^ soil, and 6.0‍ ‍mg P kg^–1^ soil, respectively, for Ci. Soils were air dried after the removal of non-soil components, such as stones and plant roots. Dry soil was crushed and sieved using a 1-mm sieve. In the experiment, soils were autoclaved twice at 121°C for 60‍ ‍min.

Inoculation experiments included one control and three treatments for each soil: PSB treatment, TCP treatment, and combination treatment. There were 5 experimental groups for both the PSB and combined treatments: A, B, F, G, and H. Each control and treatment (or each experimental group) had three replicates.

Regarding the PSB treatment, 2.5‍ ‍mL of PSB suspensions and 12.5‍ ‍mL of sterile distilled water were added to 50‍ ‍g sterilized soil. In the TCP treatment, 50‍ ‍g soil was mixed with 1% (w/w) TCP, autoclaved, and then added to 2.5‍ ‍mL of P-free NBRIP and 12.5‍ ‍mL of sterile distilled water. The soil of the combination treatment was the same as that of the TCP treatment, except for the addition of 2.5‍ ‍mL PSB suspensions instead of P-free NBRIP. As a control, 2.5‍ ‍mL of P-free NBRIP and 12.5‍ ‍mL of sterile distilled water were added to 50‍ ‍g sterilized soil. After an incubation at 28°C for 7 days, the dilution of 0.1‍ ‍g of soil was spread on NBRIP agar using the method described in above to confirm strain survival. The remaining soil was lyophilized to measure P fractions.

### Co-culture with maize seedlings

La and Ci were used in the co-culture experiment. As described in above, each soil had one control (no inoculation or fertilizer) and three treatments. One isolate showing an exceptional phosphate-solubilizing capacity was used in this experiment for the PSB and combination treatments. Each control and treatment had three replicates.

Maize (*Zea mays* L. cv. Guidan 162; Guangxi Zhaohe Seed Industry) seeds were surface sterilized once with 75% (v/v) ethanol and once with 1% (w/v) mercuric chloride for 2‍ ‍min, respectively, followed by extensive rinsing with sterile distilled water. Germination was conducted at 28°C for 24‍ ‍h under sterile conditions. Germinated seeds were transferred to culture containers containing 50‍ ‍g of soil. Five milliliters of P-free Hoagland nutrient solution (Hoagland and Arnon, 1950) and 12.5‍ ‍mL of sterile distilled water were added to each container. After an incubation at 28°C for 24 h, maize kernels were carefully removed.

In the control and TCP treatments, 2.5‍ ‍mL P-free NBRIP was added to the soil. In the PSB and combination treatments, 2.5‍ ‍mL of strain A suspension was added to the soil. Five milliliters of P-free Hoagland nutrient solution was added every other day to support plant growth. All containers were periodically watered with sterile distilled water to maintain the initial weight. After 7 days, maize shoots were harvested, dried, and weighed. After grinding the shoot to a fine powder, 0.05‍ ‍g of plant material was digested with 1‍ ‍mL of concentrated H_2_SO_4_ and H_2_O_2_. The total concentration of P in the digestion solution was assessed using the molybdenum blue method.

The dilution of 0.1‍ ‍g of soil was spread on NBRIP agar following the procedure described in above to confirm strain survival. The remaining soil was lyophilized after the removal of roots and then used for P fractionation.

### Soil P fractionation and measurement of P

The present study adopted Hedley’s sequential P fractionation method (the Hedley method; Hedley *et al.*, 1982), which is widely used to assess soil P fractionation (Yang and Post, 2011; Hou *et al.*, 2018; Xu *et al.*, 2018; Delfim *et al.*, 2020; Liu *et al.*, 2022).

Using the Hedley method, soil P fractions were examined using‍ ‍the following procedure. In the initial step of extraction, 0.5‍ ‍g soil was placed into a 50-mL centrifuge tube. Extraction was performed using 1) approximately 5‍ ‍cm^2^ anion-exchange resin (Selemion^TM^ ion exchangeable resin; AGC Engineering) and 30‍ ‍mL distilled water, 2) 30‍ ‍mL 0.5 M NaHCO_3_, 3) 30‍ ‍mL 0.1 M NaOH, and 4) 20‍ ‍mL 1 M HCl in sequence. Each extraction was shaken at 120‍ ‍rpm at 25°C for 16 h. The resin was set aside and the soil suspension was centrifuged at 6,000‍ ‍rpm for 20‍ ‍min to separate the supernatant and soil. Resin was placed into a new tube containing 20‍ ‍mL 0.5 M HCl and shaken at 120‍ ‍rpm at 25°C for 2‍ ‍h to extract resin P. Other extracts were NaHCO_3_-Pi, NaOH-Pi, and HCl P. Five milliliters of the NaHCO_3_ extract was mixed with 10‍ ‍mL 0.9‍ ‍M H_2_SO_4_ and 0.5‍ ‍g (NH_4_)_2_S_2_O_8_ was autoclaved at 120°C for 60‍ ‍min to obtain NaHCO_3_-PT (total P) (resin P and NaHCO_3_-PT were identified as labile P, which is available to plants). Five milliliters of the NaOH extract, 10‍ ‍mL 0.9 M H_2_SO_4_, and 0.6‍ ‍g (NH_4_)_2_S_2_O_8_ were autoclaved at 120°C for 90‍ ‍min to obtain NaOH-PT. NaOH-PT has been identified as moderately labile P, which is strongly held by chemisorption to the surfaces of Al and Fe oxides (Hedley *et al.*, 1982; Costa *et al.*, 2016). Po is the difference between PT and the corresponding Pi. The P concentrations of all extracts and the digested solution were quantified using the molybdenum blue method (Murphy and Riley, 1962).

### Statistical ana­lysis

All data were analyzed using SPSS (SPSS Statistics, Ver. 21.0.0.0; IBM). Values for phosphate release, the soil P fraction, and P accumulation in maize seedlings are shown as means±SE. Significant differences between means were analyzed with a one-way ANOVA (Tukey’s and Dunnett’s post hoc tests) at a significance level of 5%. Soil P fractions were subjected to a two-way ANOVA with the PSB inoculation and TCP supply. The effects of the soil type, PSB inoculation, and TCP supply on P accumulation in maize seedlings were analyzed by point-biserial correlations.

## Results

### PSB identification, assessment of phosphate release ability, and viability in soil

**Fig. 1.**
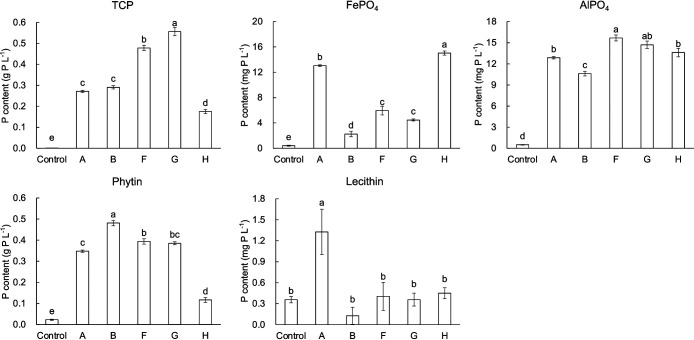
Soluble inorganic P content in culture solutions containing different P sources after a 5-days incubation with 5 PSB isolates (A, B, F, G, and H) or no bacterial isolate (Control). Different letters indicate significant differences (*P*<0.05; one-way ANOVA, Tukey, *n*=3). Error bars=SE.

Twenty-eight strains from rhizosphere soil and 7 strains from non-cultivated La were identified as being from 6 genera (data not shown). After the tests on phosphate release ability and viability in soil, 5 strains were selected for subsequent experiments (Table 1): the B strain from the rhizosphere soil of Chinese cabbage released more P from phytin than other strains; the F and G strains released more P from TCP; the A and H strains from non-cultivated La released more P from FePO_4_ (Fig. 1). These 5 strains showed viability in La and Ci after the viability test (data not shown).

### Soil inoculation experiments

After soil inoculation experiments, the inoculated strains were confirmed to have survived and bacterial growth was not observed in the TCP or control treatment (data not shown). No significant differences were noted in total P in La or Ci between the control and PSB treatments or between the TCP and combination treatments (data not shown).

In the La inoculation experiment (Table 2), all PSB treatment groups had significantly higher NaOH-Pi and lower HCl P than the control group (La-Ctrl). TCP fertilization changed the size of inorganic P fractions (*i.e.* resin P, NaHCO_3_-Pi, NaOH-Pi, and HCl P), whereas organic P fractions (NaHCO_3_-Po, NaOH-Po) remained similar. All combination treatment groups, except for LaFP, had significantly higher NaHCO_3_-Pi, NaHCO_3_-Po, and NaOH-Po than the TCP treatment (LaP), while LaAP, LaBP, and LaFP had significantly higher NaOH-Pi. The interaction between the PSB inoculation and TCP supply was significant for all P fractions, except for HCl P.

Table S1 shows changes in labile P, moderately labile P, and HCl P in the La inoculation experiment. In the La-Ctrl group, moderately labile P (23.71‍ ‍mg P kg^–1^ soil) was markedly higher than that of labile P (0.27‍ ‍mg P kg^–1^ soil). When TCP was added, the increase in moderately labile P (19.72‍ ‍mg P kg^–1^ soil) was greater than that in labile P (0.67‍ ‍mg P kg^–1^ soil). Following the PSB inoculation, increases in moderately labile P were also greater than those in labile P in both the PSB and combination treatment groups.

In the Ci inoculation experiment (Table 3), all PSB treatment groups had significantly higher resin P and NaHCO_3_-Pi than the Ci-Ctrl group. TCP supply had significantly different resin P, NaHCO_3_-Pi, and HCl P, whereas organic P fractions were unaffected, similar to LaP. All combination treatment groups had significantly higher resin P than the CiP group. The interaction between the PSB inoculation and TCP supply was significant for resin P, NaHCO_3_-Pi, NaHCO_3_-Po, and NaOH-Po, whereas NaOH-Pi and HCl P were unaffected.

As shown in Table S2, the pool sizes of labile P and moderately labile P in the Ci-Ctrl group were similar: 6.84 and 5.13‍ ‍mg P kg^–1^ soil, respectively. TCP supply increased labile P by 1.74‍ ‍mg kg^–1^ soil, whereas no increase was noted in moderately labile P. Following the PSB inoculation, increases in labile P were greater than those in moderately labile P in both the PSB and combination treatment groups, except for CiGP.

Pearson’s correlation ana­lysis of the two soils with TCP supply (TCP and combined treatments) and without TCP supply (control and PSB treatments) showed no correlation between labile P and HCl P (data not shown).

### Co-culture of PSB with maize seedlings

Strain A was used in the co-culture experiment because it caused higher labile P concentrations than the other strains in both La and Ci under TCP fertilized conditions. After the culture, the strain was confirmed to have survived, and no significant differences were observed in total P in La or Ci between the control and PSB treatments or between the TCP and combination treatments (data not shown).

In the co-culture (Table 4), La treated with PSB isolate A (LaA) showed differences in all fractions, except for NaHCO_3_-Po, from uninoculated control soil (La-Ctrl), while LaAP (relative to LaP) significantly changed all fractions, except for HCl P; CiA (relative to Ci-Ctrl) significantly changed all fractions, except for NaOH-Po, while CiAP (relative to CiP) significantly changed all fractions, except for NaOH-Po and HCl P. TCP supply significantly increased all fractions, except for NaOH-Po, in LaP, whereas it increased all fractions, except for NaOH-Pi and NaOH-Po, in CiP. The interaction between the PSB inoculation and TCP supply was significant for resin P, NaHCO_3_-Pi, and NaOH-Pi in La and for labile P (resin P, NaHCO_3_-Pi, and NaHCO_3_-Po) in Ci.

As shown in Table S3, all treatments increased moderately labile P more than labile P in La, whereas all treatments increased labile P more than moderately labile P in Ci.

**Fig. 2.**
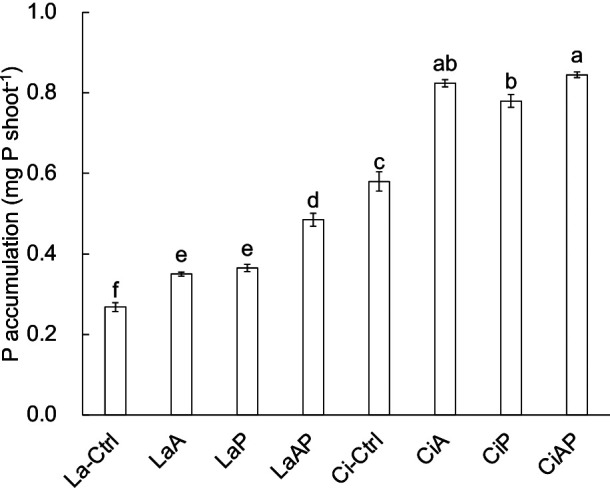
P accumulation in 7-day-old maize seedlings grown on acidic Lateritic red earths (La) or alkaline Cinnamon soils (Ci). Soils were inoculated with PSB strain A (A), fertilized with TCP (P), or treated with both PSB strain A and fertilizer (AP). Different letters indicate significant differences (*P*<0.05; one-way ANOVA, Tukey, *n*=3). Error bar=SE.

In terms of P accumulation in maize seedlings (Fig. 2), LaA and CiA were higher than La-Ctrl and Ci-Ctrl, respectively. LaP and CiP were also higher than La-Ctrl and Ci-Ctrl, respectively, LaAP was higher than La-Ctrl, LaA, and LaP, and CiAP was higher than Ci-Ctrl and CiP. No significant differences were observed between LaA and LaP or between CiA and CiP.

In La, the PSB inoculation increased P accumulation by 0.082‍ ‍mg at a rate of approximately 31%; TCP supply increased P accumulation by 0.097‍ ‍mg at a rate of approximately 36%; and the combination treatment increased P accumulation by 0.217‍ ‍mg at a rate of approximately 81%. The absolute increase and rate of increase in LaAP were higher than those in LaA. In Ci, the PSB inoculation increased P accumulation by 0.244‍ ‍mg at a rate of approximately 42%; TCP supply increased P accumulation by 0.200‍ ‍mg at a rate of approximately 35%; and the combination treatment increased P accumulation by 0.265‍ ‍mg at a rate of approximately 46%. The absolute increase and rate of increase in CiAP were similar to those in CiA.

## Discussion

### PSB and their phosphate release ability

In the present study, PSB were isolated with NBRIP, which contains TCP as the sole P source and effectively isolates PSB with high potential to solubilize TCP, but with low potential for mobilization from FePO_4_ and AlPO_4_ (Chung *et al.*, 2005; Jiang *et al.*, 2020). However, some of the PSB isolated from unplanted La as well as rhizosphere soil exhibited activity for mobilization not only from TCP, but also from FePO_4_ and AlPO_4_, which may be attributed to distinct mechanisms of P release (Zhu *et al.*, 2009; Wang *et al.*, 2014). The 5 PSB isolates used in the present study were closely related to genera that reportedly have the capacity to release P from soils (Lee *et al.*, 2016; Liu *et al.*, 2019; Safirzadeh *et al.*, 2019; Liu *et al.*, 2022). Soil inoculation experiments indicated that the 5 PSB isolated affected phosphate availability from soils with different properties (Tables 2 and 3). Furthermore, the present results showed that the efficiency of these PSB at releasing soil HCl P was inconsistent with their efficiency at releasing P from TCP in shake flasks. For example, F and G released more P from TCP than the other 3 strains (Fig. 1), but did not induce a greater decrease in HCl P than the other strains in the inoculation experiment (Tables S1 and S2). In addition to distinct mechanisms of P release, another reason may be that the environment provided by the culture medium in shake flasks markedly differed from soil. The buffering capacity of soils has been shown to limit the solubilization of soil phosphates by microorganisms (Cabala-Rosand and Wild, 1982; Gyaneshwar *et al.*, 1998); PSB organic acid production was affected by the different nitrogen and carbon conditions of soil (Cuningham and Kuiak, 1992). Previous studies also demonstrated that PSB with high solubility to TCP in the medium did not increase P accumulation in plants (Poonguzhali *et al.*, 2008; Collavino *et al.*, 2010). Therefore, it was considered unreliable to use the solubility of PSB for TCP in media to estimate the release of P from soil and the promotion of plant growth by PSB (Bashan *et al.*, 2013). This study also showed that it is not necessarily reliable to predict the release ability of PSB to soil P by the release ability to TCP.

### Relationships between P mobilization by PSB and soil types

Soil inoculation experiments revealed that although different strains may cause different P fraction changes, changes in the same soil were consistent. Regarding La, which is a soil with pH 5.0–5.5 and high PRP, its P fractions are dominated by moderately labile P. The inoculation of PSB resulted in a significant increase in moderately labile P as the main response, regardless of the supply of TCP, while a small amount of released P was transferred to the labile P pool. Delfim *et al.* (2020) also reported that changes in the P pools of Andisol and Ultisol with pH 5.5 and 5.8 by *Bacillus thuringiensis* significantly increased NaOH-Pi levels, which is consistent with the present results. In Ci at pH 8.0 with a high HCl P content, PSB mainly increased labile P. Therefore, the changes in P fractions caused by PSB may be dominated by the soil type. In other words, the different phosphate release abilities of PSB led to different changes in P fractions, whereas the changes observed in the same soil were consistently in the same direction.

### Effects of the PSB co-culture on maize seedlings

The promotive effects of PSB on plant P uptake and biomass have been widely reported (Biswas *et al.*, 2022; Dasila *et al.*, 2022; Liu *et al.*, 2022; Sabra and Ahmed, 2022). In the present study, strain A also promoted P accumulation in maize seedlings.

The present results indicated that strain A increased P accumulation in maize seedlings in both soils, independent of supplementation with TCP. Furthermore, P accumulation in maize seedlings was higher with strain A combined with TCP supply than with strain A inoculated alone in La, but not in Ci. Overall, the accumulation of and absolute increase in P in maize seedlings were lower in La than in Ci, while those in CiAP and CiA were the highest. In terms of the rate of increase, LaA was lower than CiA, while LaAP was markedly higher than CiAP. Therefore, the combination of PSB and TCP in La significantly optimized planting effects; however, in Ci, the mobilization of soil P using PSB showed promising results.

The results of the point-biserial correlation ana­lysis indicated the significant positive effects of soil types, the PSB inoculation, and TCP supply on P accumulation in maize seedlings (Table S4), and the correlation of soil type was greater than that of PSB inoculation and TCP supply. Nevertheless, the selectivity of the soil type in agricultural production is minimal. Therefore, PSB and P fertilizer may be an effective means to increase crop yield.

## Conclusions

The present study examined the effects of PSB isolates on soil P fractions in La and Ci soils and revealed distinct changes in the P fraction caused by PSB in both soil types. Furthermore, the results obtained showed that an inoculation with PSB strain A (cf. *Enterobacter chuandaensis*) promoted P accumulation in maize seedlings in soil with and without TCP fertilization. The present results suggest that the efficiency of a microbial strain at mobilizing soil P differs with soil types (La and Ci). These differences may be partly attributed to the effects of original soil P fractionation. P was assigned to different fractions during the conversion process. The diversity of global soils may require distinct P fertilization strategies (Mengel, 1997). The results of the present study support this view. La, which is rich in iron and aluminum oxides, is more likely to bind P to the moderately labile P fraction, leading to low labile P conditions.

As reported by Barrow (2022), long-term fertilized soils have already accumulated large amounts of P. PSB are an effective means to mobilize P accumulated in soils. Further studies on P release ability in different soils may contribute to the more efficient use of PSB.

## Citation

Long, H., and Wasaki, J. (2023) Effects of Phosphate-solubilizing Bacteria on Soil Phosphorus Fractions and Supply to Maize Seedlings Grown in Lateritic Red Earths and Cinnamon Soils. *Microbes Environ ***38**: ME22075.

https://doi.org/10.1264/jsme2.ME22075

## Supplementary Material

Supplementary Material

## Figures and Tables

**Table 1.** Overview of PSB strains used in experiments

Strain name	Source	Closest relatives	Similarity (%)	Classification	NCBI number
A	Lateritic red earths	*Enterobacter chuandaensis*	99.44	Bacteria;Proteobacteria;Gammaproteobacteria;Enterobacterales;Enterobacteriaceae;Enterobacter	ON778739
B	Rhizosphere soil of Chinese cabbage	*Pantoea rodasii*	99.72	Bacteria;Proteobacteria;Gammaproteobacteria;Enterobacterales;Erwiniaceae;Pantoea	ON778745
F	Rhizosphere soil of Chinese cabbage	*Klebsiella aerogenes*	92.96	Bacteria;Proteobacteria;Gammaproteobacteria;Enterobacterales;Enterobacteriaceae;Klebsiella	ON778779.1
G	Rhizosphere soil of Chinese cabbage	*Pseudomonas hunanensis*	99.86	Bacteria;Proteobacteria;Gammaproteobacteria;Pseudomonadales;Pseudomonadaceae;Pseudomonas	ON778780
H	Lateritic red earths	*Pseudomonas protegens*	98.38	Bacteria;Proteobacteria;Gammaproteobacteria;Pseudomonadales;Pseudomonadaceae;Pseudomonas	ON778778

**Table 2.** Soil P fractions (mg P kg^–1^ soil) in the La inoculation experiment

Group	Resin P	NaHCO_3_-Pi	NaHCO_3_-Po	NaOH-Pi	NaOH-Po	HCl P
La-Ctrl	0.057±0.003	0.005±0.002	0.205±0.007	14.29±0.24	9.41±0.24	0.0729±0.0108
LaA	0.075±0.003	0.029±0.004*	0.210±0.005	15.55±0.36*	9.84±0.28	0.0124±0.0032*
LaB	0.084±0.005	0.070±0.008*	0.333±0.009*	19.96±0.39*	9.09±0.31	0.0007±0.0005*
LaF	0.079±0.003	0.043±0.004*	0.210±0.005	15.44±0.25*	9.56±0.26	0.0121±0.0033*
LaG	0.062±0.007	0.056±0.005*	0.210±0.005	16.77±0.35*	9.66±0.35	0.0017±0.0008*
LaH	0.069±0.023	0.011±0.002	0.224±0.006	16.62±0.27*	9.57±0.38	0.0210±0.0049*
LaP	0.468±0.016*	0.263±0.008*	0.212±0.006	34.00±0.38*	9.43±0.23	452±6*
LaAP	1.051±0.022*	0.605±0.009*	0.288±0.006*	43.00±0.32*	10.78±0.19*	432±4
LaBP	0.457±0.012	0.490±0.019*	0.280±0.005*	40.65±0.22*	11.39±0.23*	437±9
LaFP	0.506±0.016	0.266±0.007	0.216±0.009	35.36±0.29*	9.56±0.27	448±8
LaGP	0.542±0.009*	0.335±0.013*	0.284±0.006*	34.86±0.30	12.68±0.22*	451±6
LaHP	0.445±0.009	0.456±0.015*	0.350±0.008*	34.03±0.33	12.71±0.32*	443±9
PSB	***	***	***	***	***	—
TCP	***	***	***	***	***	***
PSB * TCP	***	***	***	***	***	—

Values represent the mean of three replicates±SE (standard errors). Significant differences among means were tested with a one-way ANOVA (Dunnett. Use La-Ctrl as the control category for LaA~LaH and LaP; use LaP for LaAP~LaHP). La-Ctrl: control, La with P-free NBRIP added; LaA~LaH: PSB treatment, La with A~H suspension inoculation; LaP: TCP treatment, La with TCP supply and P-free NBRIP added; LaAP~LaHP: Combination treatment, La with A~H suspension inoculation and TCP supply. PSB, TCP, and PSB*TCP: Two-way ANOVA for the factors of the PSB inoculation, TCP supply, and the interaction of the PSB inoculation ×TCP supply. *** *P*<0.001, ** *P*<0.01, * *P*<0.05, — *P*≥0.05.

**Table 3.** Soil P fractions (mg P kg^–1^ soil) in the Ci inoculation experiment

Group	Resin P	NaHCO_3_-Pi	NaHCO_3_-Po	NaOH-Pi	NaOH-Po	HCl P
Ci-Ctrl	1.76±0.01	4.13±0.01	0.95±0.01	2.38±0.01	2.76±0.01	307±2
CiA	3.05±0.01*	4.49±0.04*	1.44±0.02*	2.39±0.01	3.41±0.01*	300±3
CiB	3.98±0.01*	4.36±0.05*	1.54±0.01*	2.38±0.01	2.73±0.02	299±2
CiF	2.39±0.01*	5.84±0.11*	1.78±0.05*	2.28±0.02*	2.83±0.02*	300±4
CiG	1.92±0.01*	4.97±0.07*	1.34±0.11*	2.44±0.02*	2.74±0.01	304±3
CiH	2.43±0.01*	4.89±0.05*	1.12±0.02	2.52±0.01*	2.87±0.01*	302±3
CiP	2.02±0.02*	5.62±0.02*	0.94±0.03	2.38±0.02	2.76±0.03	1800±9*
CiAP	4.10±0.01*	6.17±0.02*	1.43±0.02*	2.44±0.05	3.69±0.01*	1787±7
CiBP	4.10±0.01*	5.82±0.06	0.72±0.02*	2.49±0.04	2.80±0.02	1790±6
CiFP	2.82±0.07*	6.17±0.06*	1.78±0.06*	2.36±0.03	3.01±0.02*	1792±9
CiGP	2.84±0.02*	5.03±0.03*	1.36±0.09*	2.56±0.03*	3.38±0.04*	1793±9
CiHP	2.84±0.01*	6.05±0.14*	1.12±0.07	2.53±0.04*	2.99±0.01*	1793±10
PSB	***	***	***	***	***	—
TCP	***	***	***	***	***	***
PSB * TCP	***	***	***	—	***	—

Values represent the mean of three replicates±SE (standard errors). Significant differences were assessed using a one-way ANOVA (Dunnett. Use Ci-Ctrl as the control category for CiA~CiH and CiP; use CiP for CiAP~CiHP). Ci-Ctrl: control, Ci with P-free NBRIP added; CiA~CiH: PSB treatment, Ci with A~H suspension inoculation; CiP: TCP treatment, Ci with TCP supply and P-free NBRIP added; CiAP~CiHP: Combination treatment, Ci with A~H suspension inoculation and TCP supply. PSB, TCP, and PSB×TCP: Two-way ANOVA for the factors of the PSB inoculation, TCP supply, and the interaction of PSB inoculation ×TCP supply. *** *P*<0.001, ** *P*<0.01, * *P*<0.05, — *P*≥0.05.

**Table 4.** Soil P fractions (mg P kg^–1^ soil) in co-cultured La and Ci

Group	Resin P	NaHCO_3_-Pi	NaHCO_3_-Po	NaOH-Pi	NaOH-Po	HCl P
La-Ctrl	0.027±0.003d	0.023±0.002d	0.212±0.004c	14.79±0.26d	9.43±0.23c	0.065±0.006b
LaA	0.061±0.003c	0.068±0.004c	0.226±0.004c	18.00±0.23c	10.99±0.20b	0.007±0.002c
LaP	0.564±0.006b	0.361±0.006b	0.256±0.006b	40.99±0.34b	10.11±0.23c	436±3a
LaAP	1.174±0.015a	0.649±0.021a	0.292±0.011a	47.00±0.24a	12.10±0.30a	427±2a
PSB	***	***	***	***	***	*
TCP	***	***	***	***	***	***
TCP * PSB	***	***	—	***	—	*
Ci-Ctrl	1.86±0.02d	3.82±0.08d	1.72±0.05c	2.05±0.01b	4.36±0.02b	306±4b
CiA	2.47±0.03c	5.11±0.03c	2.19±0.05b	2.39±0.01a	4.39±0.02ab	291±2c
CiP	3.89±0.09b	6.09±0.03b	2.10±0.05b	2.06±0.04b	4.30±0.09ab	1704±8a
CiAP	4.20±0.07a	6.92±0.08a	3.32±0.05a	2.40±0.02a	4.49±0.04a	1701±9a
PSB	***	***	***	***	*	—
TCP	***	***	***	—	—	***
TCP * PSB	*	***	***	—	—	—

Values represent the mean of three replicates±SE (standard errors). Significant differences in a column under each group are indicated by different letters (P≤0.05). Significance was analyzed with a one-way ANOVA (Games-Howell). La-Ctrl: control, La with P-free NBRIP added; LaA: PSB treatment, La with A suspension inoculation; LaP: TCP treatment, La with TCP supply and P-free NBRIP added; LaAP: Combination treatment, La with A suspension inoculation and TCP supply. Ci-Ctrl: control, Ci with P-free NBRIP added; CiA: PSB treatment, Ci with A suspension inoculation; CiP: TCP treatment, Ci with TCP supply and P-free NBRIP added; CiAP: Combination treatment, Ci with A~H suspension inoculation and TCP supply. PSB, TCP, and PSB*TCP: Two-way ANOVA for the factors of the PSB inoculation, TCP supply, and the interaction of PSB inoculation ×TCP supply. *** *P*<0.001, ** *P*<0.01, * *P*<0.05, — *P*≥0.05.
