# Supplementary material for: Effects of Phosphate-solubilizing Bacteria on Soil Phosphorus Fractions and Supply to Maize Seedlings Grown in Lateritic Red Earths and Cinnamon Soils
Source: Microbes Environ. 2023 May 23;38(2):ME22075. doi: 10.1264/jsme2.ME22075 (PMC10308236; doi:10.1264/jsme2.ME22075)
Supplement: Supplementary file 1 — Supplementary Material [file 38_22075_s1.pdf]

Table S1. Changes of labile P, moderately labile P and HCl P in La inoculation experiment (mg-P kg^{-1} soil)

Group	Labile P	Labile P Increase		Moderately labile P	Moderately labile P Increase		HCl P decrease
La-Ctrl	0.27			23.71			
		Relative to La-Ctrl			Relative to La-Ctrl		Relative to La-Ctrl
LaA	0.31	0.04		25.39	1.68		0.06
LaB	0.49	0.22		29.05	5.34		0.07
LaF	0.33	0.06		25.00	1.29		0.06
LaG	0.33	0.06		26.43	2.72		0.07
LaH	0.30	0.03		26.20	2.49		0.05
LaP	0.94	0.67		43.43	19.72		
			Relative to LaP			Relative to LaP	Relative to LaP
LaAP	1.94	1.67	1.00	53.78	30.07	10.35	20
LaBP	1.23	0.96	0.29	52.04	28.33	8.61	15
LaFP	0.99	0.72	0.05	44.92	21.21	1.49	4
LaGP	1.16	0.89	0.22	47.54	23.83	4.11	1
LaHP	1.25	0.98	0.31	46.75	23.04	3.32	9

La-Ctrl: control, La with P-free NBRIP adding; LaA ~ LaH: PSB treatment, La with A ~ H strains inoculation；LaP: TCP treatment, La with TCP supply；LaAP ~ LaHP: Combination treatment, La with A~H strains inoculation and TCP supply; labile P: sum of Resin P, $NaHCO_3$-Pi and $NaHCO_3$-Po in Table 2; moderately labile P: sum of NaOH-Pi and NaOH-Po in Table 2.

Table S2. Changes of labile P, moderately labile P and HCl P in Ci inoculation experiment (mg-P kg-1 soil)

Group	Labile P	Labile P Increase		Moderately labile P	Moderately labile P Increase		HCl P decrease
Ci-Ctrl	6.84			5.13			
		Relative to Ci-Ctrl			Relative to Ci-Ctrl		Relative to Ci-Ctrl
CiA	8.98	2.14		5.80	0.67		7
CiB	9.88	3.04		5.11	-0.02		8
CiF	10.01	3.17		5.11	-0.02		7
CiG	8.23	1.39		5.18	0.05		3
CiH	8.44	1.60		5.38	0.25		5
CiP	8.58	1.74		5.14	0.01		
			Relative to CiP			Relative to CiP	Relative to CiP
CiAP	11.7	4.86	3.12	6.14	1.00	0.99	13
CiBP	10.63	3.79	2.05	5.29	0.15	0.14	10
CiFP	10.77	3.93	2.19	5.37	0.23	0.22	8
CiGP	9.22	2.38	0.64	5.94	0.80	0.79	7
CiHP	10.01	3.17	1.43	5.52	0.38	0.37	7

Ci-Ctrl: control, Ci with P-free NBRIP adding; CiA ~ CiH: PSB treatment, Ci with A ~ H strains inoculation; CiP: TCP treatment, Ci with TCP supply; CiAP ~ CiHP: Combination treatment, Ci with A~H strains inoculation and TCP supply; labile P: sum of Resin P, $NaHCO_3$-Pi and $NaHCO_3$-Po in Table 3; moderately labile P: sum of NaOH-Pi and NaOH-Po in Table 3.

Table S3. Changes of labile P, moderately labile P and HCl P in co-culture experiment (mg-P kg^{-1} soil)

Group	Labile P	Increase	Moderately labile P	Increase
La-Ctrl	0.26	Relative to La-Ctrl	24.23	Relative to La-Ctrl
LaA	0.36	0.10	29.00	4.77
LaP	1.18	0.92	51.10	26.87
LaAP	2.11	1.85	59.10	34.87
Ci-Ctrl	7.40	Relative to Ci-Ctrl	6.41	Relative to Ci-Ctrl
CiA	9.77	2.37	6.78	0.37
CiP	12.08	4.68	6.36	-0.05
CiAP	14.44	7.04	6.88	0.47

La-Ctrl, Ci-Ctrl: control; LaA, CiA: PSB treatment; LaP, CiP: TCP treatment; LaAP, CiAP: combination treatment; labile P: sum of Resin P, $NaHCO_3$-Pi and $NaHCO_3$-Po in Table 4; moderately labile P: sum of NaOH-Pi and NaOH-Po in Table 4.

Table S4 Point-Biserial Correlation analysis

	Soil type	PSB inoculation	TCP supply
Maize seedling P accumulation	0.859**	0.281**	0.249**

Maize seedling P accumulation: continuous variable.
Soil type, PSB inoculation and TCP supply: categorical variable, assign “0” to “La”, “no PSB inoculation” and “no TCP supply”; assign “1” to “Ci”, “PSB inoculation” and “TCP supply”.

** $P < 0.01$
